# Supplementary material for: Unravelling the Puzzle of Anthranoid Metabolism in Living Plant Cells Using Spectral Imaging Coupled to Mass Spectrometry
Source: Metabolites. 2021 Aug 25;11(9):571. doi: 10.3390/metabo11090571 (PMC8472718; doi:10.3390/metabo11090571)
Supplement: Supplementary file 1 [file metabolites-11-00571-s001.zip › metabolites-1350856-supplementary.pdf]

metabolites

MDPI

Article

Unravelling the puzzle of anthranoids metabolism in living plant cells using spectral imaging coupled to mass spectrometry

Quentin Chevalier *, Jean-Baptiste Gallé, Nicolas Wasser, Valérie Mazan, Claire Villette, Jérôme Mutterer, Maria M. Elustondo, Nicolas Girard, Mourad Elhabiri, Hubert Schaller, Andréa Hemmerlin and Catherine Vonthron-Sénécheau

***Correspondence: qchevalier67@gmail.com**

Supplementary Materials

This PDF file includes:

1. Supplemental Figures S1 to S18
2. Supplemental Tables S1 to S5
3. Supplemental Data References

1. Supplemental Figures

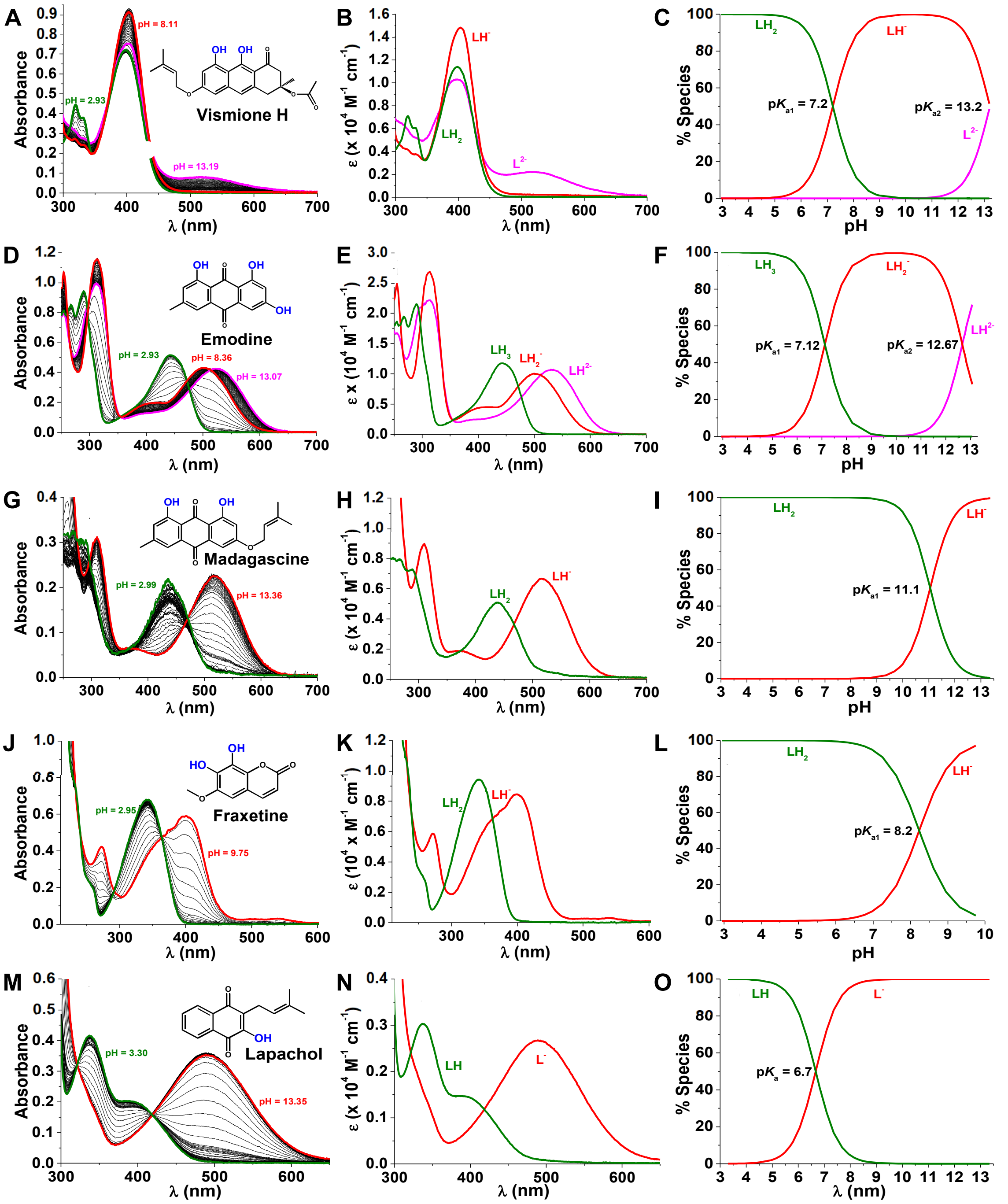

Figure S1. Influence of pH on absorption properties of studied phenolic compounds. (left) UV-Vis. absorption spectrophotometric titrations versus pH of most of the compounds considered in this work, (middle) electronic absorption spectra and (right) distribution diagrams as a function of pH of their protonated species. **(A, B, C)** 63.2 μM Vismione H; **(D,E,F)** 43.8 μM Emodin Emo, **(G,H,I)** 35 μM Madagascine Mad, **(J, K, L)** 70.9 μM Fraxetin Fra and **(M, N, O)** 136 μM Lapachol Lap. Solvent: EtOH/water 1:1 v/v; I = 0.1 M (NaCl); T = 25°C. Under our experimental conditions, all pK_{a1} have been characterized and were calculated to be below 9 except for Mad for which pK_{a1} was measured to be 11.1 ± 0.1 (i.e., β-hydroxy-ketone unit stabilized by hydrogen bong). The pK_a value determined for Lap (pK_a = 6.7 ± 0.1) was found to be in excellent agreement with data reported elsewhere (pK_a = 6.31 ± 0.03) under the same experimental conditions [1]. Emo and VH both display a second ionizable site for which the corresponding pK_{a2} values were measured to be 12.7 ± 0.2 and 13.2 ± 0.2, respectively (Table S1). For Emo, the second protonation site could be assigned to the 8-hydroxy group, while the first one was easily assigned to the 3-hydroxy unit. These data are in agreement with pK_a values (pK_{a1}= 8.0 ± 0.1 and pK_{a2} = 10.9 ± 0.2.) reported for Emo in MeOH/water 3:1 [2]. The second pK_a value of Fra could not be

determined under our experimental conditions. Consistent is the fact that Fra was most likely degraded with a putative lactone breakage at pH > 9-10. For Emo and Mad, the other pK_a values were estimated to be above 13 and could not be determined under our experimental conditions. Finally, the pK_a values of Qui have been reported to be 8.5 and 10.65 in EtOH/water 1:1 [3] and were not re-determined in this study.

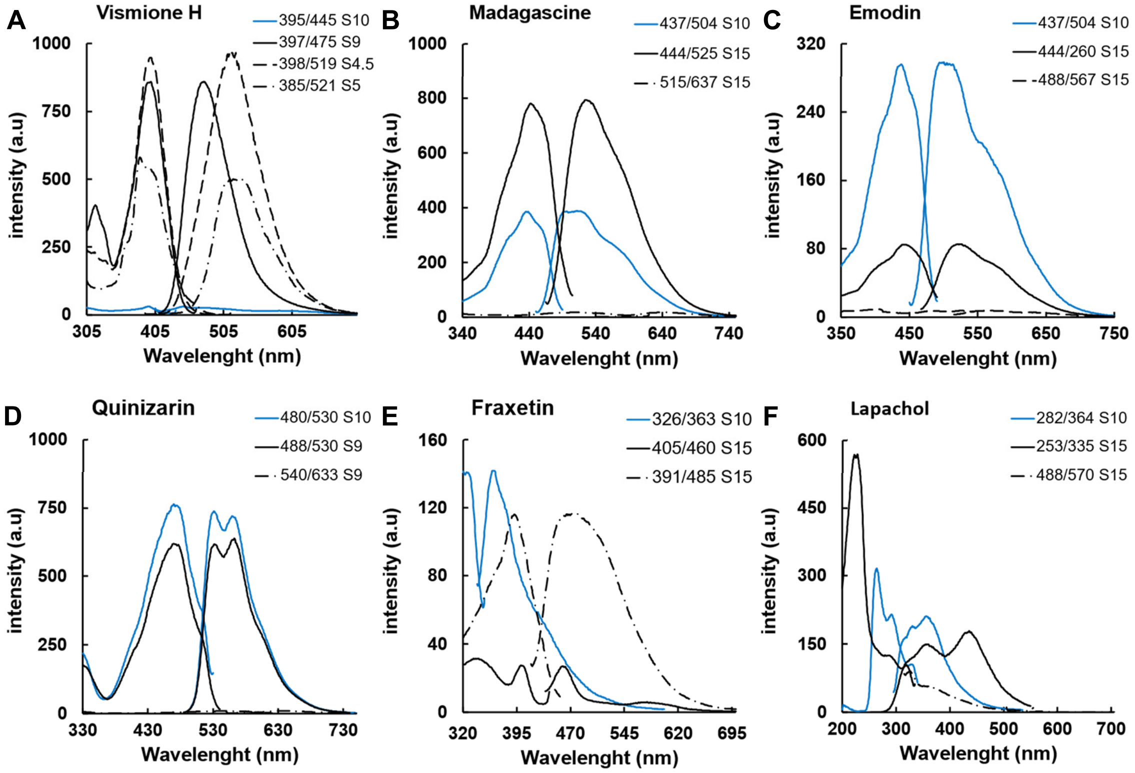

Figure S2. Fluorescence emission/excitation spectra of studied compounds in solution at different pH. **(A)** vismione H VH 2.6 μM**, (B)** emodin Emo 5.5 μM, **(C)** madagascine Mad 3 μM, **(D)** quinizarin Qui 4.2 μM, **(E)** fraxetin Fra 4.8 μM, **(F)** lapachol Lap 4.12 μM in different solvents: EtOAc (blue) and ethanolic solution (black), at pH 2 (solid line), pH 10 $Na_2B_4O_7$ (dashed line) and pH 12 (dashed dotted line). S value corresponds to excitation/emission bandwidths of the spectrofluorimeter.

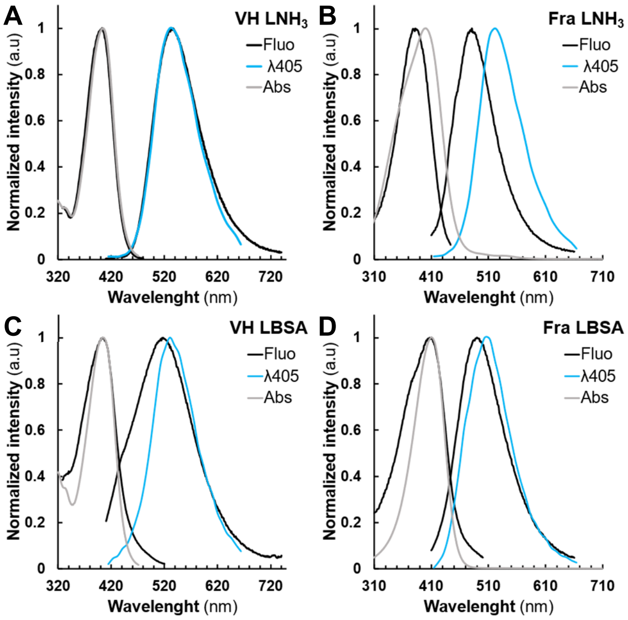

Figure S3. Normalized fluorescence excitation/emission and absorption spectra of LNH_4^+ and LBSA complexes. **(A)** ammonium complex LNH_4^+ species of vismione H VH, **(C)** BSA complex LBSA species of VH and **(B)** ammonium complex LNH_4^+ species of fraxetin Fra, **(D)** BSA complex LBSA species of fraxetin Fra in 0.01M NH_4HCO_3 saline ethanolic solutions or water containing 300 μM BSA. To note 26 μM VH and 48 μM Fra were used to measure fluorescence of LBSA complexes by spectral imaging, while spectra of LNH_4^+ species were obtained at 1 mM. Excitation and emission spectra obtained from the spectrofluorimetric analysis (black), absorption spectra obtained from the UV-Vis analysis (grey) and spectral imaging at λ_{405} (blue) and/or λ_{488} (green) settings.

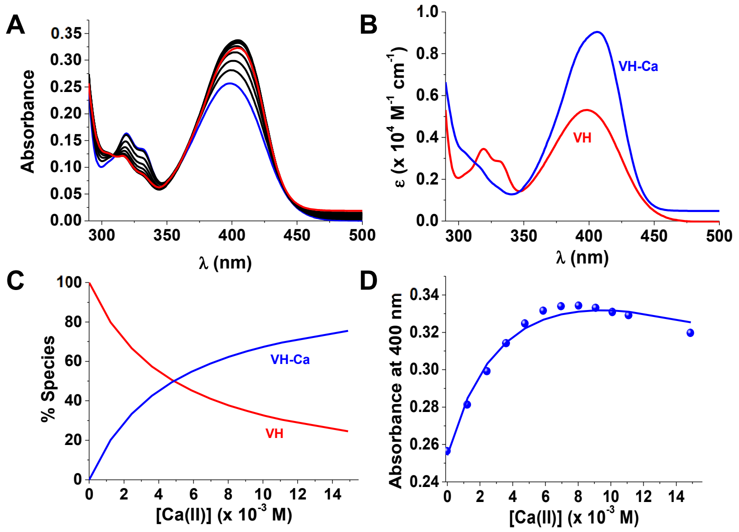

Figure S4. UV-Vis. absorption titration of vismione H by Ca(II). **(A)** Absorption spectral variation of VH as a function of [Ca(II)]. **(B)** Electronic absorption spectra of VH and its Ca(II) complex. **(C)** Species distribution diagrams as a function of [Ca(II)]. **(D)** Absorbance at 400 nm versus [Ca(II)] compared to the calculated data (solid line). Solvent: EtOH/water 1:1 v/v, I = 0.1 M NaCl; T = 25°C; [VH] = 26 μM.

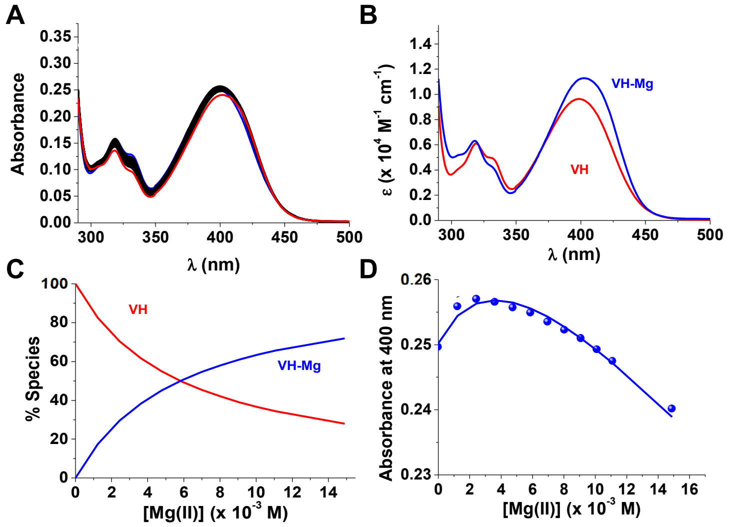

Figure S5. UV-Vis. absorption titration of vismione H by Mg(II). **(A)** Absorption spectral variation of VH as a function of [Mg(II)]. **(B)** Electronic absorption spectra of VH and its Mg(II) complex. **(C)** Species distribution diagrams as a function of [Mg(II)]. **(D)** Absorbance at 400 nm versus [Mg(II)] compared to the calculated data (solid line). Solvent: EtOH/water 1:1 v/v, I = 0.1 M NaCl; T = 25°C; [VH] = 26 μM.

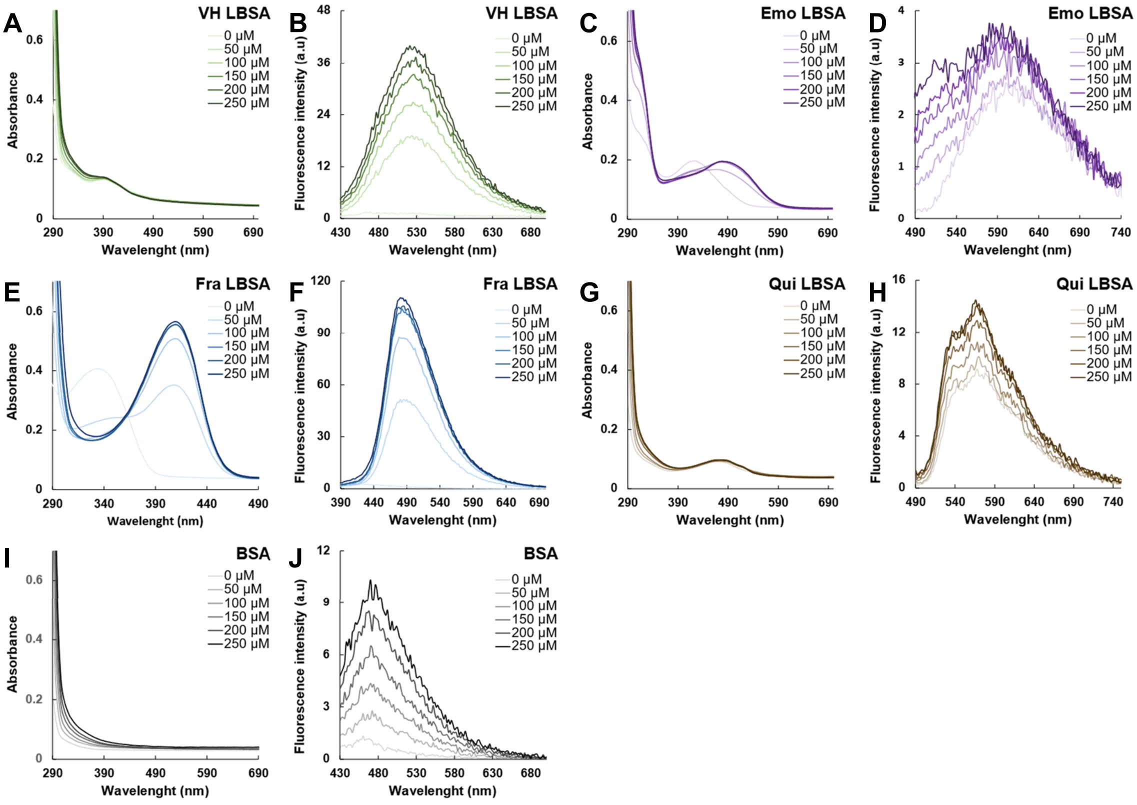

Figure S6. Absorption and fluorescence emission spectra of anthranoids and fraxetin LBSA complexes. BSA concentration ranged from 0 to 250 µM with **(A, B**) 26 µM vismione H VH, **(C, D)** 55 µM emodin Emo, **(E, F)** 48 µM fraxetin Fra, **(G, H)** 41.6 µM quinizarin Qui and **(I, J)** BSA alone. An important bathochromic shift of the absorption bands was observed for Emo LBSA and Fra LBSA, while VH and Qui absorption exhibit weak to no variations in the presence of BSA. As far as fluorescence is concerned, the emission of Fra and VH were significantly altered by the addition of BSA (important hyperchromic shift) while those of Qui and Emo were weakly altered. These data suggest that Fra, VH and Emo interacted with terminal amines (or ammoniums) of the protein with alterations of the absorption and emission properties. It is noteworthy that these spectral variations are similar to those observed when studying the protonation properties thus suggesting proton exchange and electrostatic interactions. Complexation reaction was performed using pure crystalline BSA powder > 98 % (Sigma-Aldrich, Saint-Louis USA) solubilized at 100 mg/mL in distilled water and stock solution of pure compounds at 0.1 mg/mL freshly prepared in EtOH. Then, appropriated volumes of BSA and pure compounds stock solutions were distributed in a Molecular Probes® 96-well microplate for fluorescence-based assays (Sigma-Aldrich, Saint-Louis USA) and distilled water was added to a final volume of 200 µL. The absorption and fluorescence emission were recorded using a Varioskan™ Flash Spectral Scanning Multimode Reader v 4.00.53 (Thermo Fisher Scientific, Waltham USA) monitored by SkanIt Software 2.4.5 RE for Varioskan Flash. The absorption spectra were recorded from 270 to 750 nm for all compounds and fluorescence emission scan settings were adjusted for VH (λ_{Ex} 400 nm, λ_{Em} 425-750 nm), Fra (λ_{Ex} 360 nm, λ_{Em} 380-700 nm), Emo (λ_{Ex} 450 nm, λ_{Em} 470-700 nm) and Qui (λ_{Ex} 470 nm, λ_{Em} 490-700 nm) with a scanning wavelengths step size of 2 nm, measurement time of 100 ms/step and a bandwidth of 5 nm.

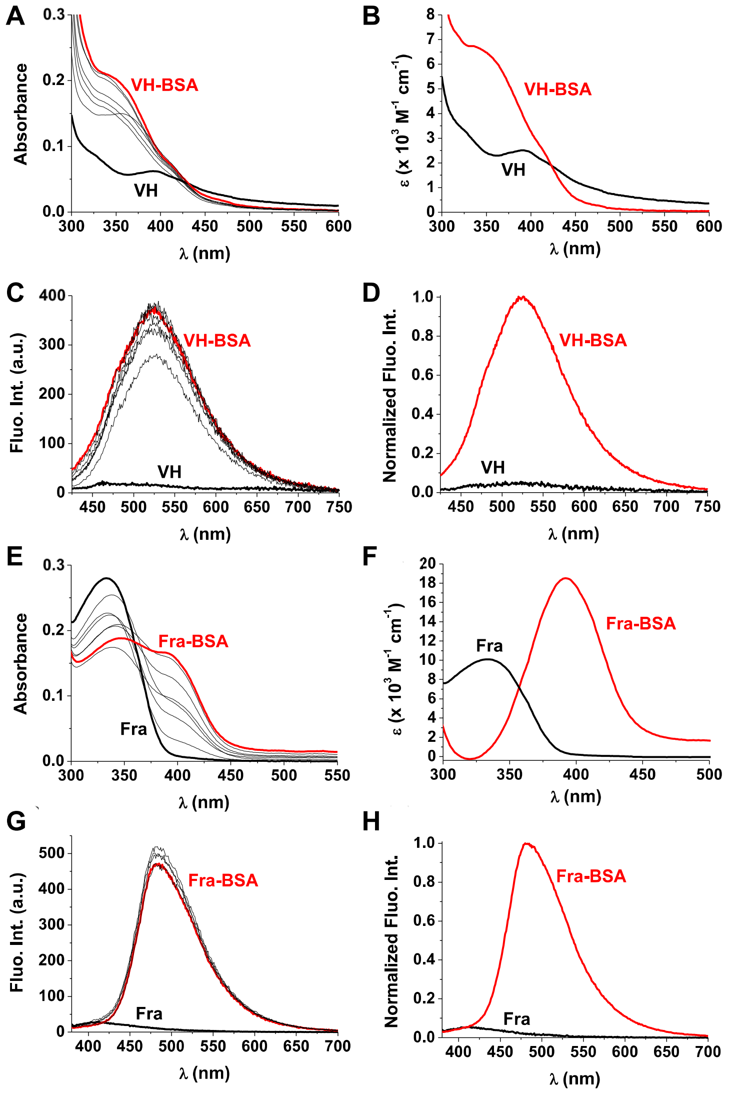

Figure S7. Corrected Absorption and fluorescence properties of fraxetin and vismione LBSA complexes. **(A, B, E and F)** Absorption and **(C, D, G, H)** fluorescence emission spectra of LBSA complexes formed by increasing concentrations of BSA from 0 to 262 μM with, **(A-D)** 26 μM vismione H VH and **(E-H)** 48 μM fraxetin Fra. **(A and E)** Absorption spectrophotometric spectra, **(B and F)** electronic absorption spectra**, (C and G)** fluorescence emission spectra and **(D and H)** reconstituted normalized emission spectra. The stability constants of the VH- and Fra-BSA complexes resulted from a statistical processing of the spectrophotometric data sets with the Specfit program. The absorption and fluorescence emission were recorded using a Varioskan™ Flash Spectral Scanning Multimode Reader v 4.00.53 (Thermo Fisher Scientific, Waltham USA) monitored by SkanIt Software 2.4.5 RE for Varioskan Flash. The absorption spectra were recorded from 270 to 750 nm for all compounds and fluorescence emission scan settings were adjusted for VH (λ_{Ex} 400 nm, λ_{Em} 425-750 nm), Fra (λ_{Ex} 360 nm, λ_{Em} 380-700 nm) with a scanning wavelengths step size of 2 nm, measurement time of 100 ms/step and a bandwidth of 5 nm.

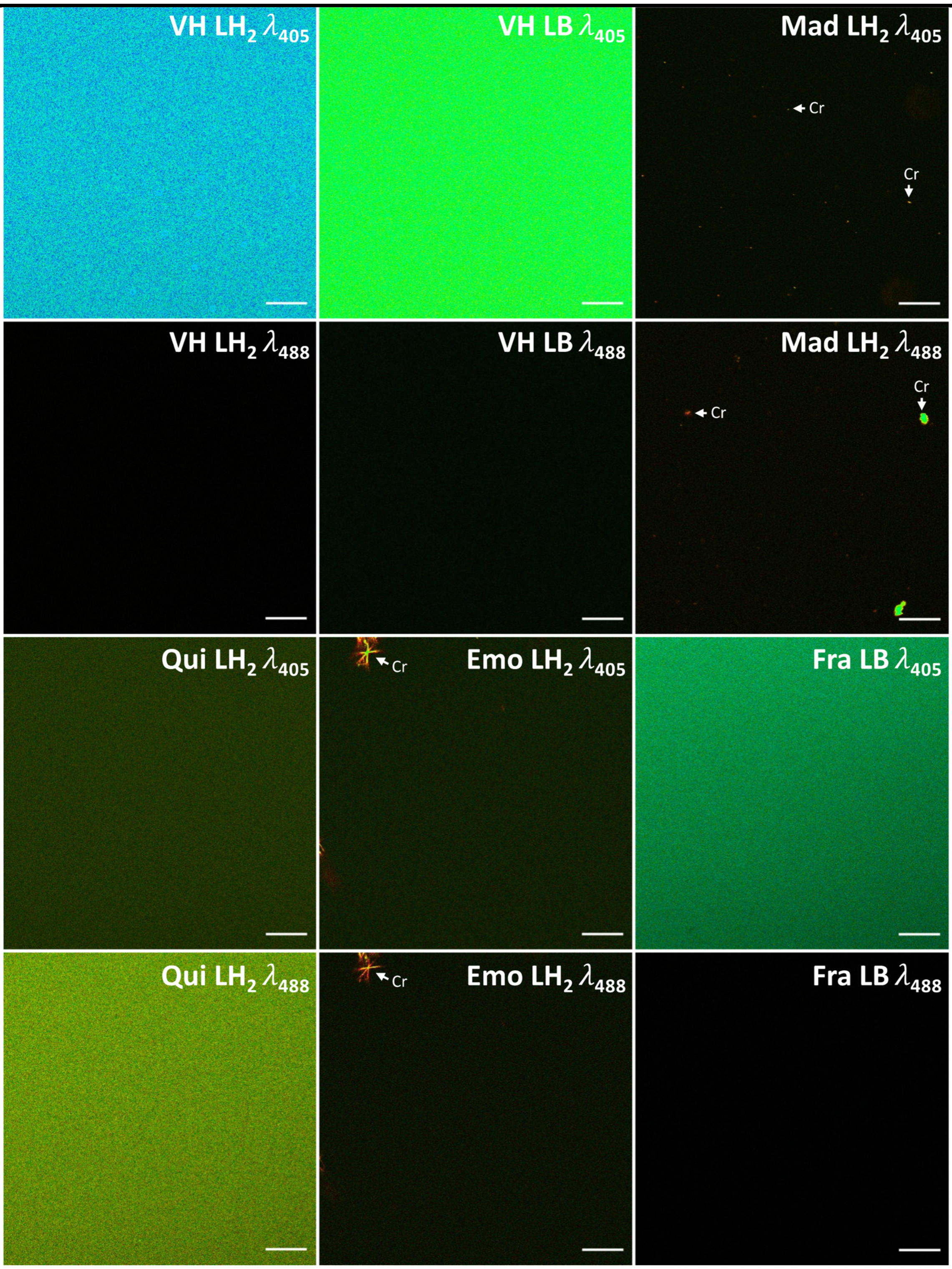

Figure S8. Images of fluorescent species from studied phenolic compounds in solution observed by SImaging. Observation of fluorescence by SImaging at λ_{Ex} 405 nm, λ_{Em} 415-664 nm (λ_{405}) and λ_{Ex} 488 nm, λ_{Em} 495-663 nm (λ_{488}) settings for vismione H VH (neutral LH_2 and boron complex LB species), madagascine Mad (neutral LH_2 species), quinizarin Qui (neutral LH_2 species), emodin Emo (neutral LH_3 species) and fraxetin Fra (boron complex LB species) in saline ethanolic solution at pH 2 (neutral species) and in pH 10 $Na_2B_4O_7$ (boron complex species). Bars = 20 µm

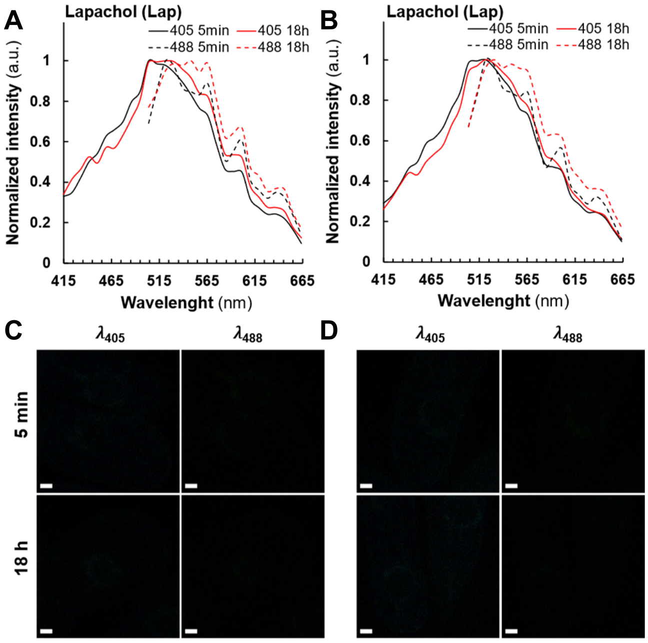

Figure S9. Normalized average spectra and images from SImaging analysis of lapachol treated cells. Analysis was performed at λ_{Ex} 405 nm, λ_{Em} 415-664 nm (λ_{405}, solid line) and λ_{Ex} 488 nm, λ_{Em} 495-663 nm (λ_{488}, dashed line) with BY-2 cells treated 5 min and 18 h by **(A)** 25 µM and **(B)** 50 µM of lapachol Lap. The absence of significative fluorescence is clearly observable by black images. Bars = 20 µm

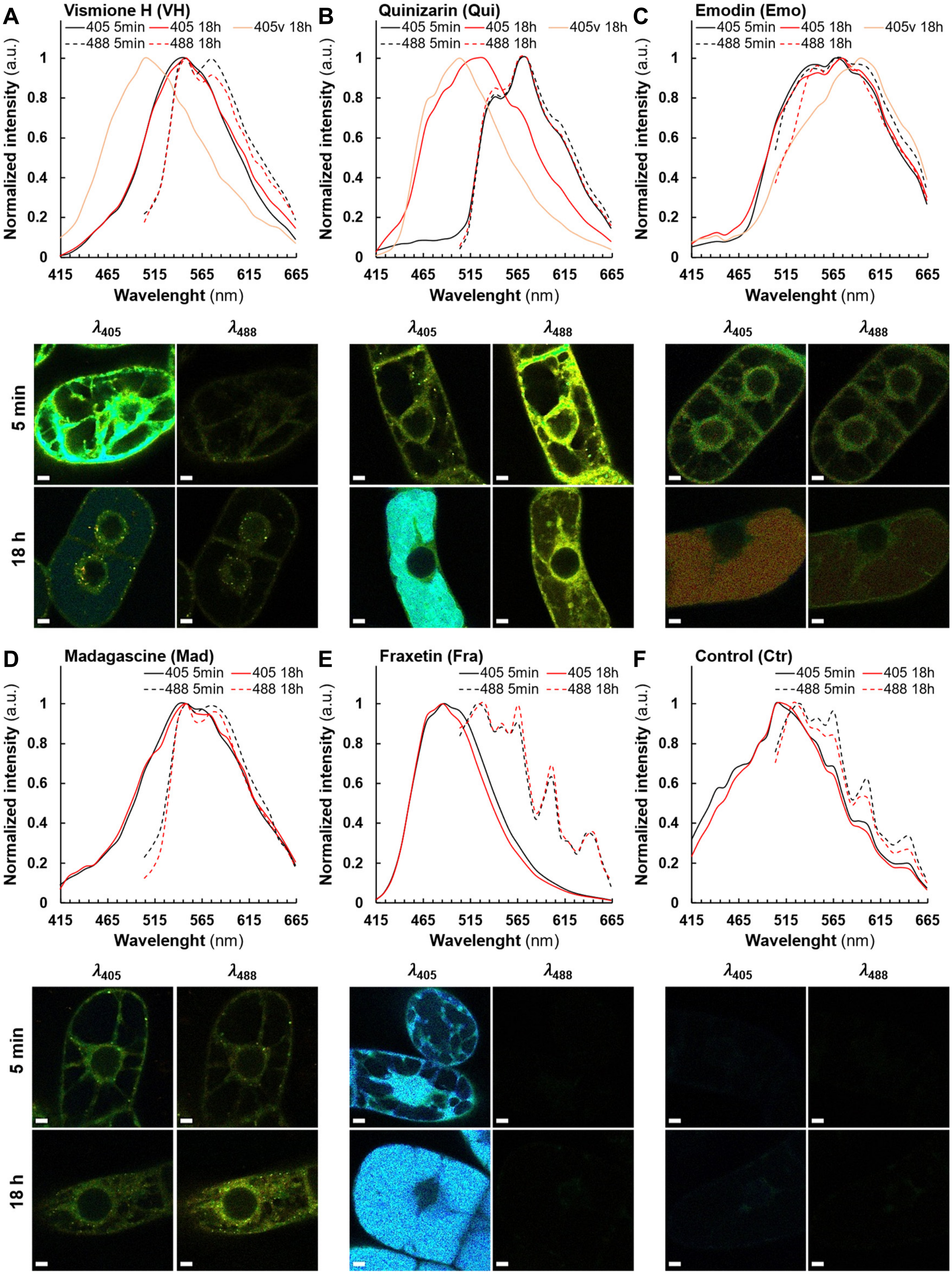

Figure S10. Fluorescence spectra and images of BY-2 cells treated by 50 µM of phenolic compounds prior to observation using SImaging. Normalized fluorescence average spectra and lambda view images from SImaging analysis at λ_{405} (solid line) and λ_{488} (dashed line) of BY-2 cells treated for 5 min and 18 h with 50 µM of **(A)** vismione H VH, **(B)** quinizarin Qui, **(C)** emodin Emo**, (D)** madagascine Mad, **(E)** fraxetin Fra and **(F)** the negative control. Spectra observed after 5 minutes (black), after 18 h (red) and in the vacuole after 18 h (red light). Spectra observed in the primary vacuole at λ_{405} after 18 h are specified with a "v" after the labels if another fluorescence was observed in the cytoplasm. Bars = 20 µm.

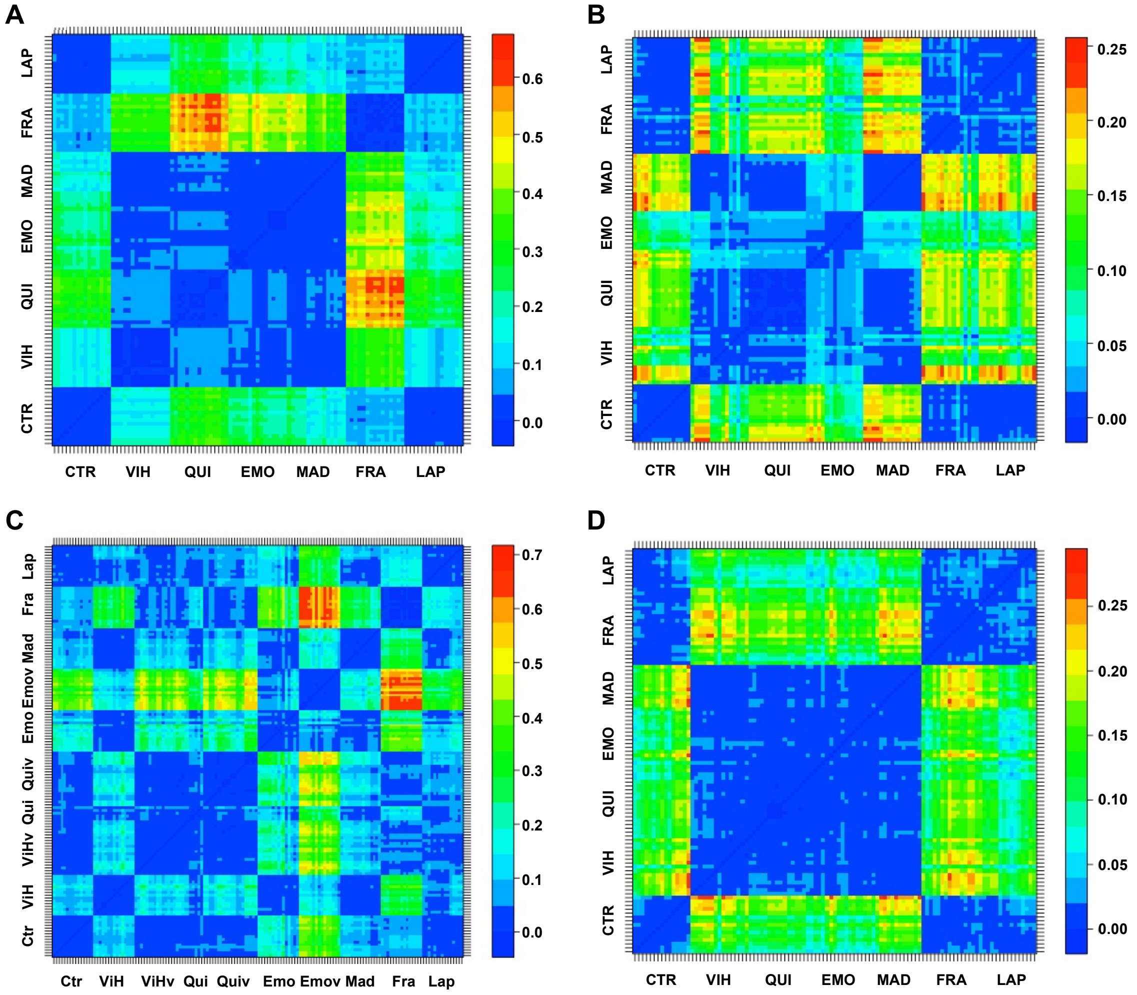

Figure S11. Distance matrices of normalized average spectra obtained by SImaging analysis of BY-2 cells treated or not with 25 µM of phenolic compounds. **(A, B)** 5 min and **(C, D)** 18 h of treatment. Spectra observed at **(A, C)** λ_{405} nm and **(B, D)** λ_{488} nm in control cells (CTR) and treated cells: vismione H (VH), quinizarin (Qui), Emodin (Emo), madagascine (Mad), fraxetin (Fra) and lapachol (Lap). Spectra observed in the primary vacuole at λ_{405} after 18 h are specified with a "v" after the labels if another fluorescence was observed in the cytoplasm. High correlation between spectra values (SS_{res} = 0, blue), low correlation (SS_{res} = 1, red)

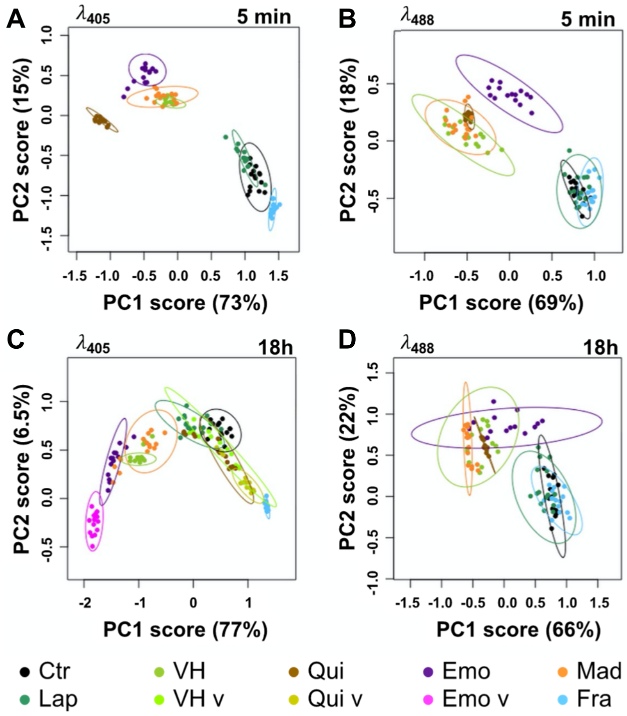

Figure S12. PCA of normalized average spectra obtained by SImaging analysis of BY-2 cells treated or not (Ctr) with 50 μM of phenolic compounds. **(A, B)** Differences observed after 5 min treatments and **(C, D)** 18 h with vismione H VH, madagascine Mad, emodin Emo, quinizarin Qui, fraxetin Fra and lapachol Lap. Spectra observed at λ_{405} **(A, C)** and λ_{488} **(B, D)** in control and treated cells. Spectra observed in the primary vacuole at λ_{405} after 18 h are specified with a "v" after the labels if another fluorescence was observed in the cytoplasm. Ellipses are representative of qualitative differences with a $p \leq 0.05$ at the PCA analysis of normalized average spectra.

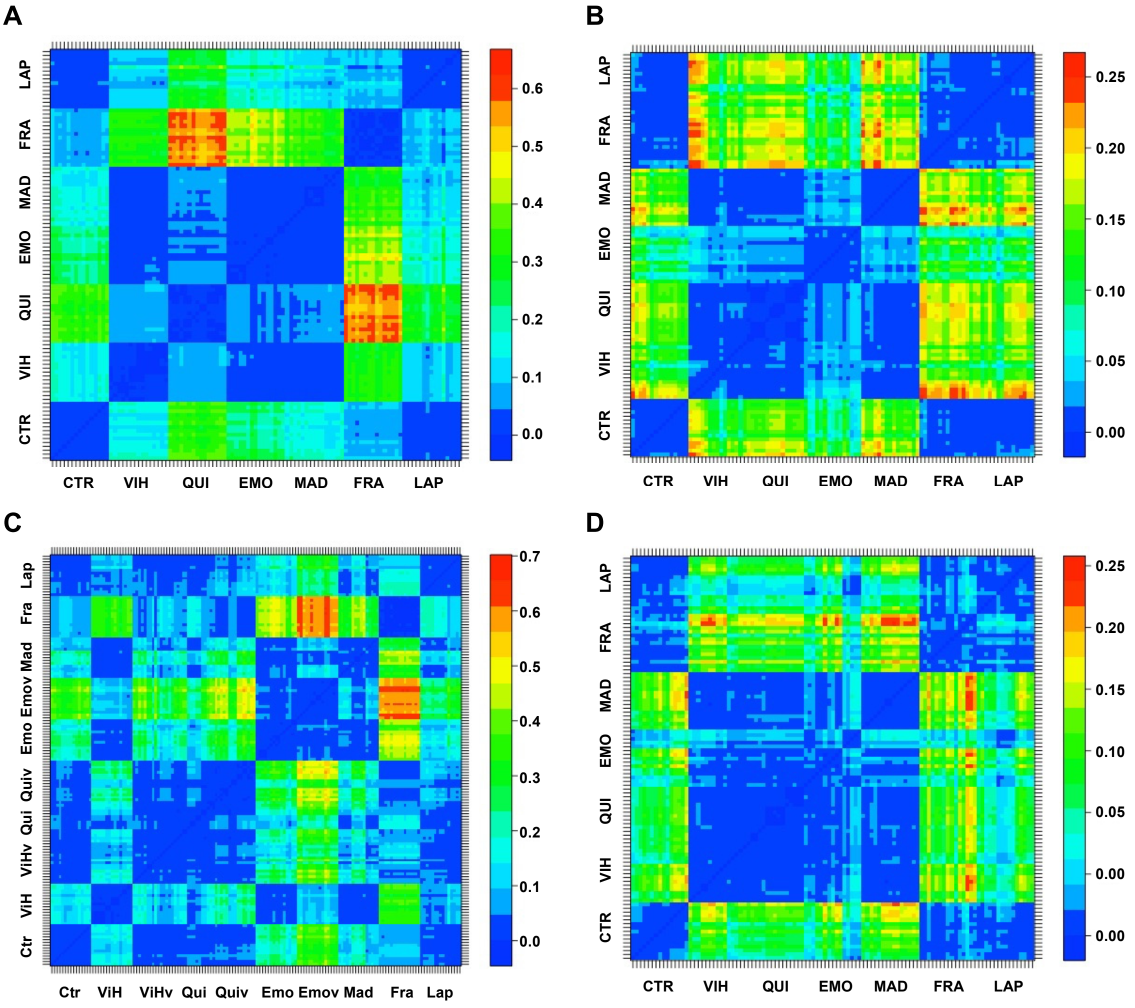

Figure S13. Distance matrices of normalized average spectra obtained by SImaging analysis of cells treated or not with 50 µM of phenolic compounds. **(A, B)** 5 min and **(C, D)** 18 h of treatment. Spectra observed at **(A, C)** λ_{405} nm and **(B, D)** λ_{488} nm in control cells (CTR) and treated cells: vismione H (VH), quinizarin (Qui), Emodin (Emo), madagascine (Mad), fraxetin (Fra) and lapachol (Lap). Spectra observed in the primary vacuole at λ_{405} after 18 h are specified with a "v" after the labels if another fluorescence was observed in the cytoplasm. High correlation between spectra values (SS_{res} = 0, blue), low correlation (SS_{res} = 1, red).

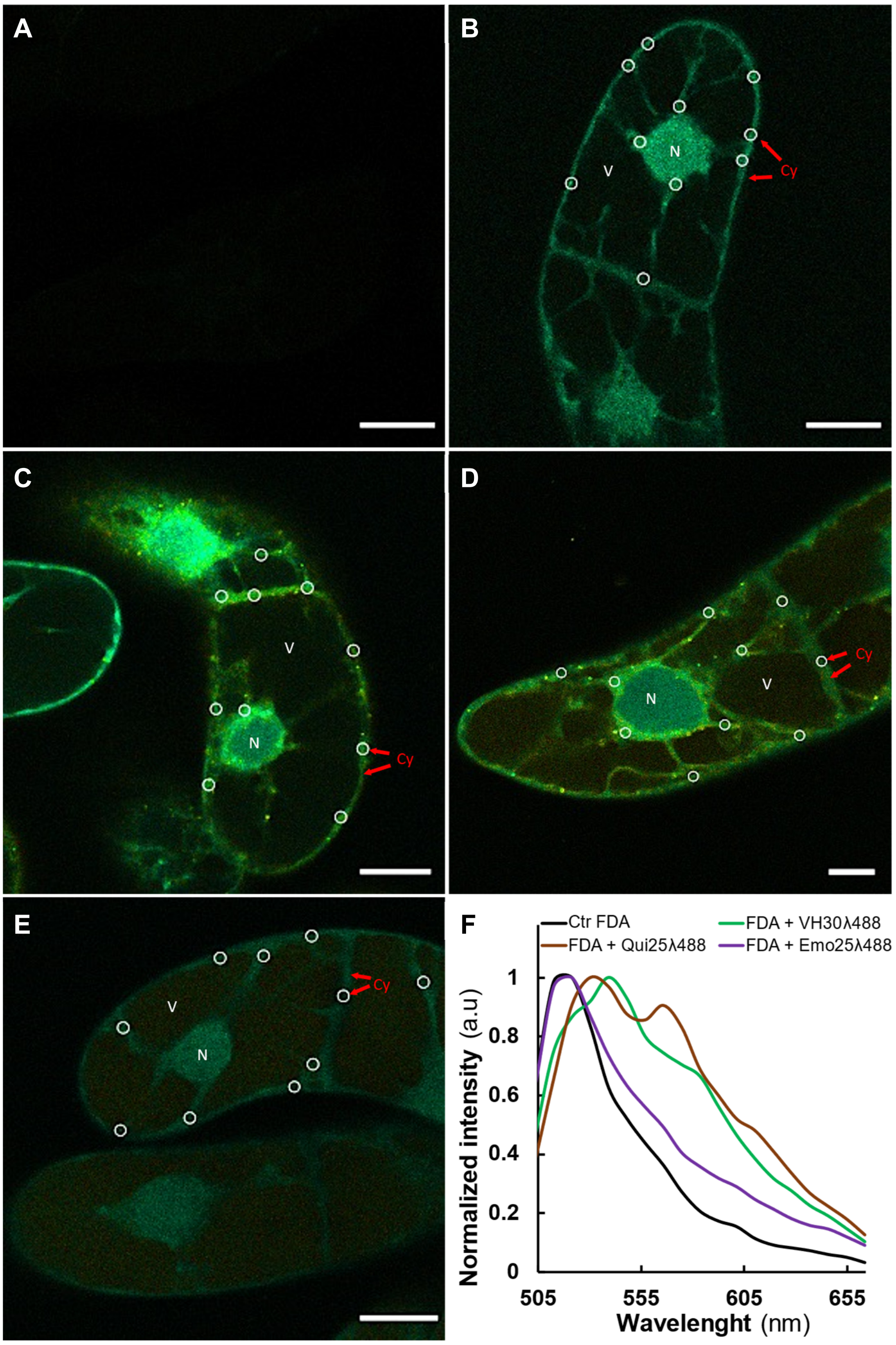

Figure S14. Anthranoids fluorescence locates in the cytoplasm of living BY-2 cells. The latter were labeled with fluorescein diacetate (FDA) viability marker after 18 h of treatment. The FDA was added to a final concentration of 75 nM in a suspension of BY-2 cells (100 µL) spotted onto a glass microscope slide prior to observation by SImaging using λ_{Ex} 488 nm (λ_{488}) and emission wavelength ranging from 498 – 664 nm. **(A)** The negative control of 7-d-old BY-2 cells without FDA labeling, **(B)** with FDA alone (Ctr FDA), or with FDA after treatments for 18 h with **(C)** 30 µM vismione H (FDA + VH30 λ_{488}), **(D)** 25 µM quinizarin (FDA + Qui25 λ_{488}), (E) 25 µM emodin (FDA + Emo25 λ_{488}). As indicated, the cytoplasm (Cy, red arrows) and the nucleus (N, white) are labeled by FDA fluorescence in living cells, while not the primary vacuole (V, white). The colocalization of FDA with the other fluorescent compounds was confirmed by analysis of fluorescence emission spectra collected in 10 different areas associated to cytoplasm of BY-2 cells (white circle). Bars = 20 µm. **(F)** Spectra recorded were normalized to 1 and averaged prior to comparison between the different conditions. Accordingly, the spectra recorded in the cytoplasm of the control with only FDA were different from those obtained in anthranoids treated cells, highlighting thus hybrid spectra as a result of colabelling between anthranoids and FDA fluorescence emission.

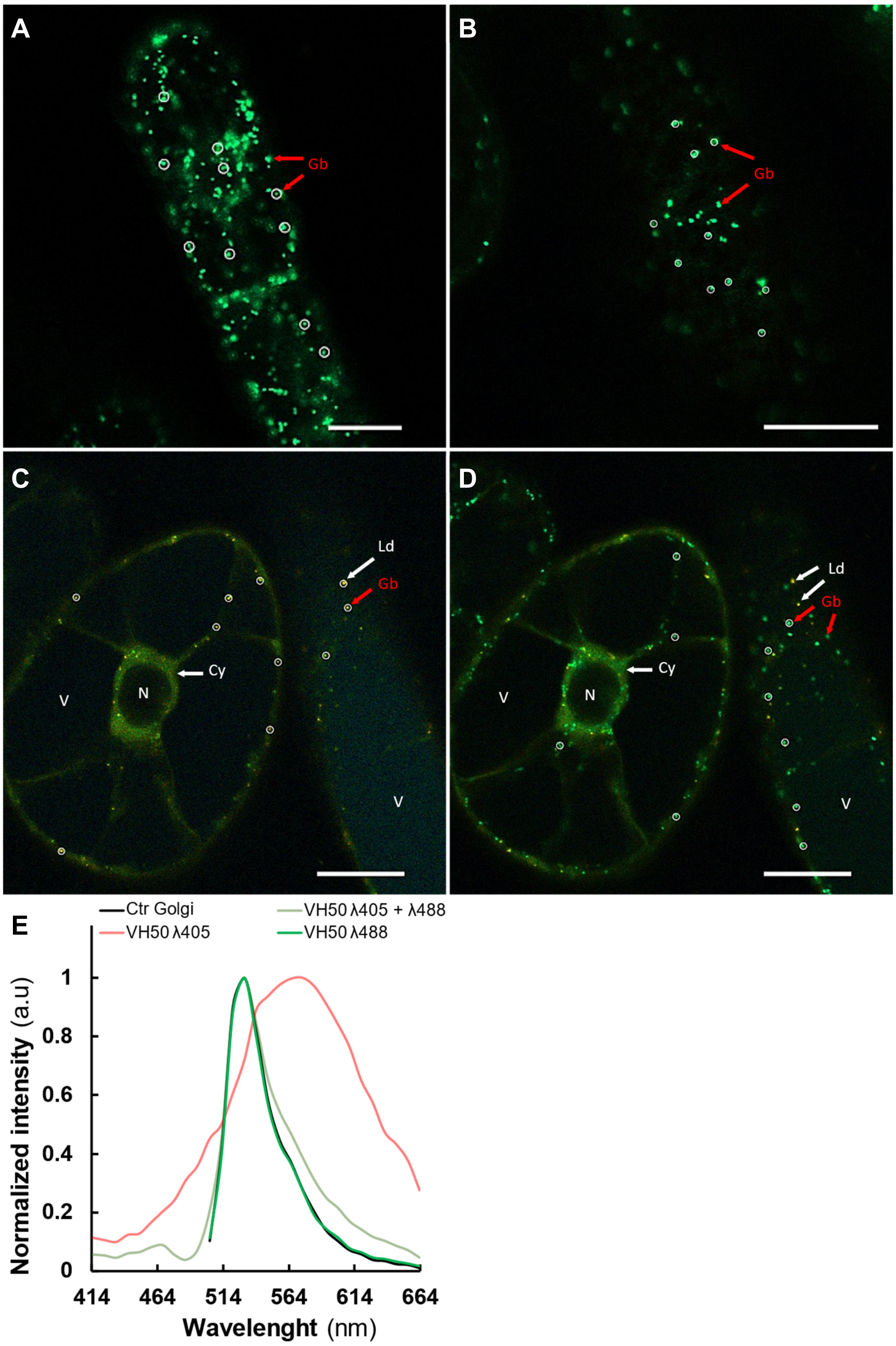

Figure S15. Vismione H (VH) and related anthraquinones fluorescence locate in the Golgi bodies of BY-2 cells. Colocalization experiments were performed with BY-2 cells expressing 35SGmMan1::eGFP fusion protein [4]. Images were acquired by SImaging with a laser power set at 5% using λ_{Ex} 488 nm (λ_{488}) and emission wavelength ranging from 498 – 664 nm, λ_{Ex} 405 nm (λ_{405}) alone or combined to λ_{Ex} 488 nm ($\lambda_{405} + \lambda_{488}$) at laser power 1% with emission wavelength ranging from 415 – 664 nm. **(A)** The negative control of 7-d-old BY-2 cells expressing 35SGmMan1::GFP alone was observed at λ_{488} (Ctr Golgi) or treated for 18 h with 50 µM VH and observed **(B)** at λ_{488} (VH50 λ_{488}), **(C)** at λ_{405} (VH50 λ_{405}) and **(D)** at $\lambda_{405} + \lambda_{488}$ (VH50 $\lambda_{405} + \lambda_{488}$). As indicated, the GmMan1::eGFP fluorescence located in the median Golgi body (Gb, red arrows), but not in subcellular compartments associated to the primary vacuole (V, white), the nucleus (N, white), the cytoplasm (Cy, white) or lipid droplets (Ld, white). Since 35SGmMan1::GFP was constitutively expressed and its fluorescence not observable under λ_{405} settings or stronger than VH related anthraquinones at λ_{488}. The settings $\lambda_{405} + \lambda_{488}$ was required to observe colocalization of both fluorescence emission. The analysis of fluorescence emission spectra collected in 10 different areas associated to GmMan1::GFP of BY-2 cells and/or vesicular bodies labeled by VH related anthraquinones (white circle). Bars = 20 µm. **(E)** Spectra recorded were normalized to 1 and averaged prior to comparison between the different conditions. Accordingly, the spectra recorded in the Golgi body at λ_{488} in Ctr Golgi and VH50 λ_{488} were similar, but differences emerged at λ_{405} in VH50 spectrum, highlighting thus hybrid spectra composed mostly of GFP fluorescence that is weakly influenced by VH related anthraquinones fluorescence. The comparison VH50 $\lambda_{405} + \lambda_{488}$ with VH50 λ_{405} obtained from identical cells at 15 sec intervals demonstrate that some Golgi body are labeled indeed, whereas other vesicular bodies are only labeled by VH related anthraquinones. Further,

colocalization experiments support that the latter are associated to lipid droplets (Figure S16). However, as 35SGmMan1::eGFP is reported to label median Golgi body, it cannot be excluded that some of them correspond to cis- or trans-Golgi compartments.

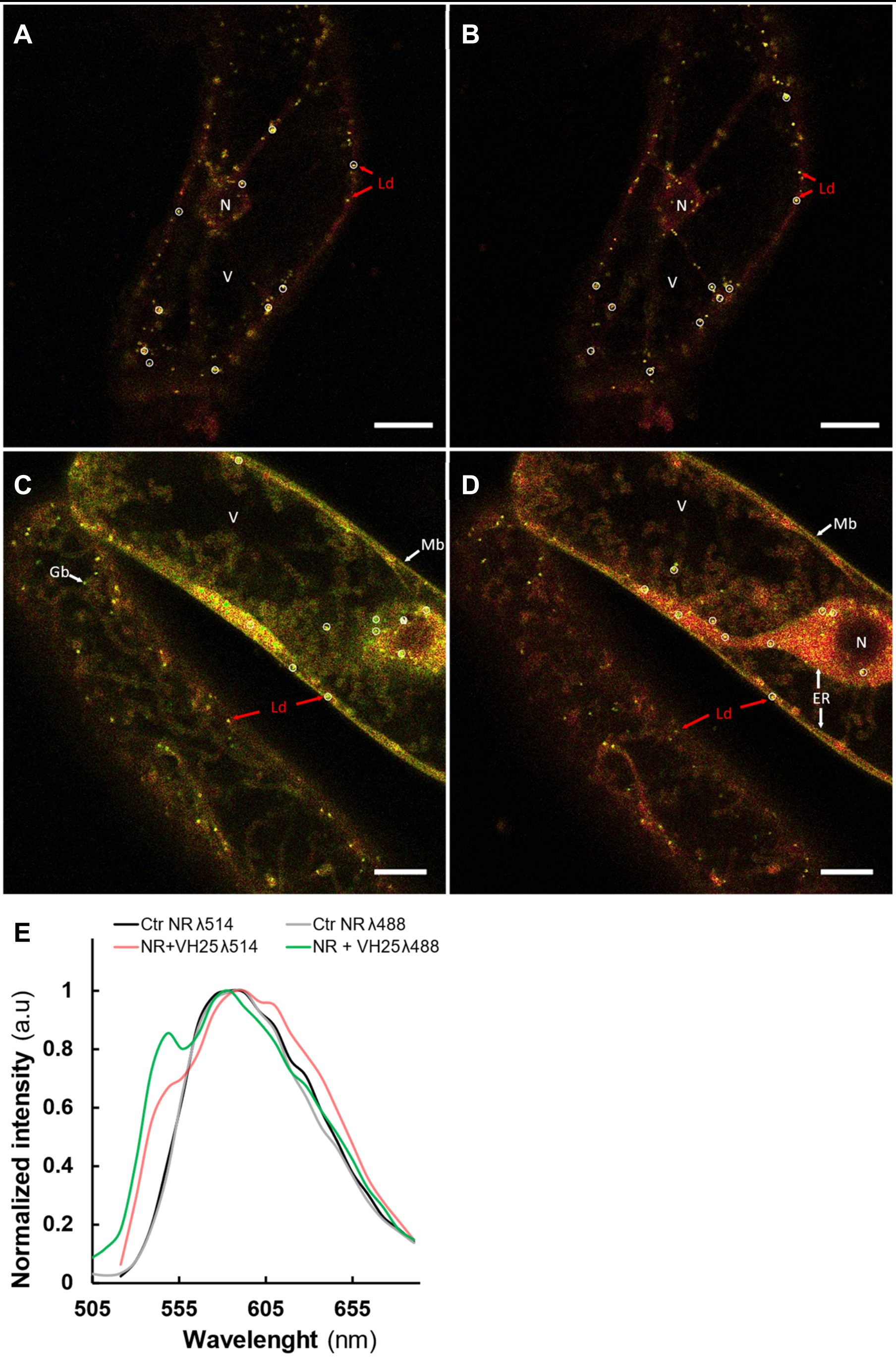

Figure S16. Vismione H (VH) related anthraquinones fluorescence locate in lipid droplets of living BY-2 cells. The latter were labeled by Nile red (NR) probe after 18 h of treatments. NR powder (Thermo Fisher, Waltham USA) was dissolved at 94 mM in EtOH stock solution and then added to a final concentration of 56 μM in a suspension of BY-2 cells (100 μL) spotted onto a glass microscope slide prior to observation by SImaging using λ_{Ex} 488 nm (λ_{488}) and emission wavelength ranging from 498 – 664 nm or λ_{Ex} 514 nm (λ_{514}) and emission wavelength ranging from 535 – 664 nm. **(A)** The negative control of 7-d-old BY-2 cells with NR labeling alone observed at λ_{488} (Ctr NR λ_{488}) and at **(B)** λ_{514} (Ctr NR λ_{514}), or with NR after treatments for 18 h with 25 μM VH and observation **(C)** at λ_{488} (NR + VH25 λ_{488}) and **(D)** at λ_{514} (NR + VH25 λ_{514}). As indicated, lipid droplets (Ld, red arrows) are labeled by a yellow-gold fluorescence as reported [5], while the endomembrane network including the endoplasmic reticulum (ER, white) is labeled by a reddish fluorescence. The colocalization of NR with the VH related anthraquinones fluorescence was confirmed by analysis of fluorescence emission spectra collected in 10 different areas associated to lipid droplets of BY-2 cells (white circle). Bars = 20 μm. (E) Spectra recorded were normalized to 1 and averaged prior to comparison between the different conditions. Accordingly, the spectra recorded in the lipid droplets of the control with only NR were different from those obtained in VH-treated cells as shown by hybrid spectra with a shoulder at 549 nm under λ_{488} and λ_{514} settings, resulting from the colabelling of VH related anthraquinones and NR fluorescence emission. Interestingly in VH treated cells, the fluorescence intensity was increased, especially in the plasma membrane (Mb, white arrows) and the ER (ER, white arrows), supporting that VH and/or related anthraquinones also locate in these subcellular compartments. Since VH is not fluorescent in hydrophobic system (Figure S2A) and the plasma membrane was not labeled with VH alone

(Figure 4A), it can be proposed that fluorescence energy transfer (FRET) between one of the VH species and NR occurred not only in plasma membrane, but also in the ER and lipid droplets promoting thus the fluorescence of NR. Finally, other fluorescent vesicular bodies were labeled by VH anthraquinones but not NR fluorescence, they were thus associated to Golgi body.

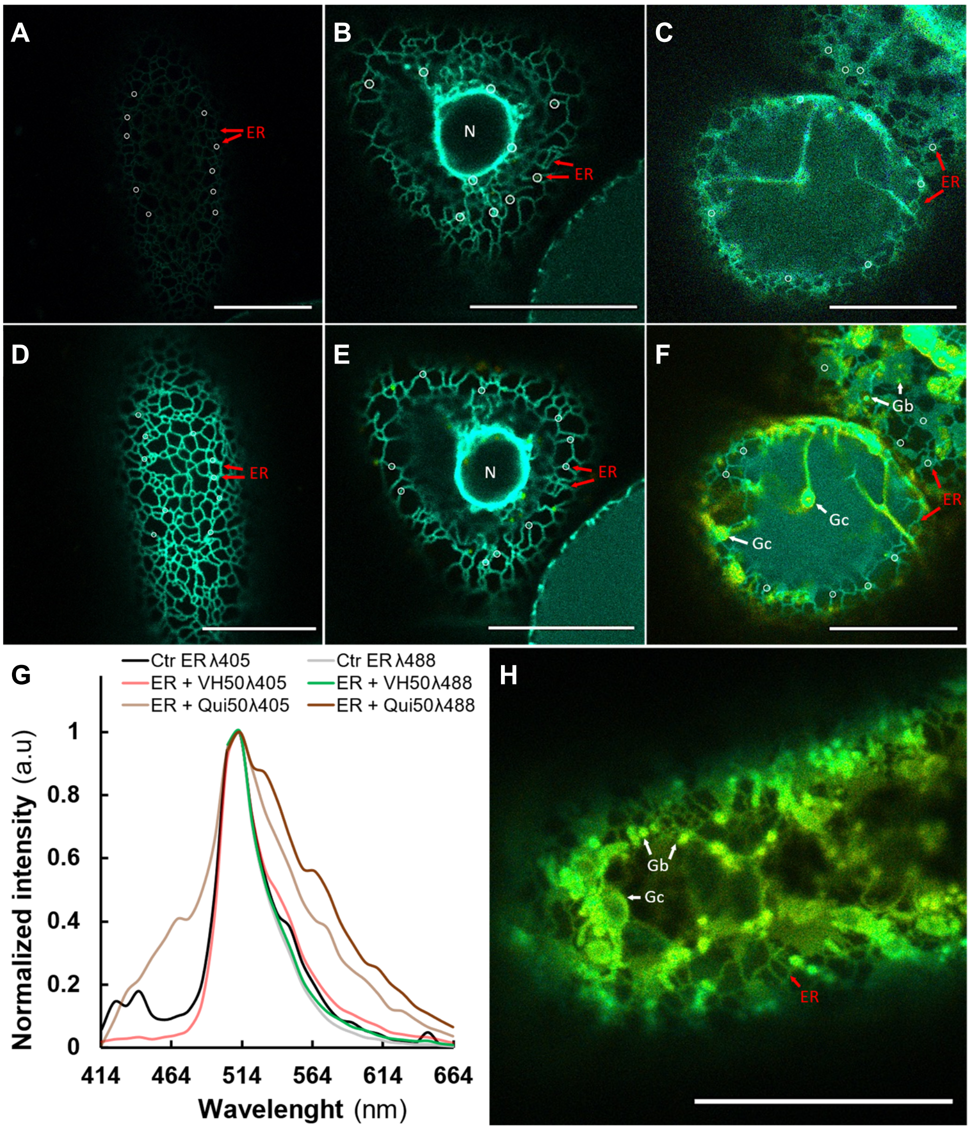

Figure S17. Anthranoids fluorescence locate in the endoplasmic reticulum (ER) of BY-2 cells. Colocalization experiments were performed with BY-2 cells expressing 35SsGFP::HDEL fusion protein [6], being located in the ER. Images were acquired by SImaging using λEx 488 nm (λ488) with emission wavelength ranging from 498 – 664 nm settings and λEx 405 nm (λ405) with emission wavelength ranging from 415 – 664 nm. The ER (ER, red arrows) was well observed at the cell surface of 7-d-old BY-2 cells expressing 35SsGFP::HDEL alone (negative control) with a very weak fluorescence at λ405 (**A**, Ctr ER λ405) in contrast to λ488 (**D**, Ctr ER λ488). In addition, same line was treated for 18 h with 50 µM vismione H (VH) and observed at λ405 (**B**, ER + VH50 λ405), λ488 (E, ER + VH50 λ488), or with 50 µM quinizarin (Qui) and observed at λ405 (**C**, ER + Qui50 λ405) and λ488 (**F**, ER + Qui50 λ488). The analysis of fluorescence was performed by collecting emission spectra in 10 different areas associated to sGFP::HDEL labeling of BY-2 cells (white circle). Bars = 20 µm. (G) Spectra recorded were then normalized to 1 and averaged prior to comparison between the different conditions. Accordingly, the spectra recorded in the Ctr ER at λ488 and λ405 were similar, as the fluorescence emission at λ405 was weaker, the quality of spectra recorded was affected by background signal and variability. Interestingly in the ER + VH50 λ405 an intense signal was observed and it corresponds to the sGFP::HDEL, indicating a colabelling with VH or/and mad anthrone in the ER. Thus, the strong fluorescence signal can be explained by FRET between VH LH2 and sGFP::HDEL being excite at λ488 (Figure 2A). Also, the ER localization of VH was confirmed by the observation at λ405 of the same reticulated structure in (H) BY-2 control cell line treated with VH 50 µM for 5 min without any expression of sGFP::HDEL. In the ER + VH50 λ405, although fluorescence observed was intense, the spectral fingerprint shows a small shoulder at 548 nm contrasting with that of ER + VH50 λ488 and may partially result from VH and/or anthraquinones as observed at λ488 settings (Figure 2 and Figure 4D).The latter was confirmed by the observation of the quinizarin fluorescence in the ER + Qui50 λ488 and ER + Qui50 λ405 with a spectral fingerprint (brown and light brown) clearly different from that of the control cells. The other subcellular structures labeled by the yellow orange quinizarin fluorescence (Figure 4) especially in the ER + Qui50 λ488 correspond to cytoplasm (Figure S14D) and Golgi body (Gb, white arrows) as small dots and likely Golgi cisternae (Gc, white arrows) being larger. (H) The observation at λ405 of the cell surface in BY-2 cells treated for 5 min with 50 µM VH showcases the cell compartments labeled by VH and/or mad anthrones associated to the ER, Golgy body and cisternae, according to all our colocalization experiments.

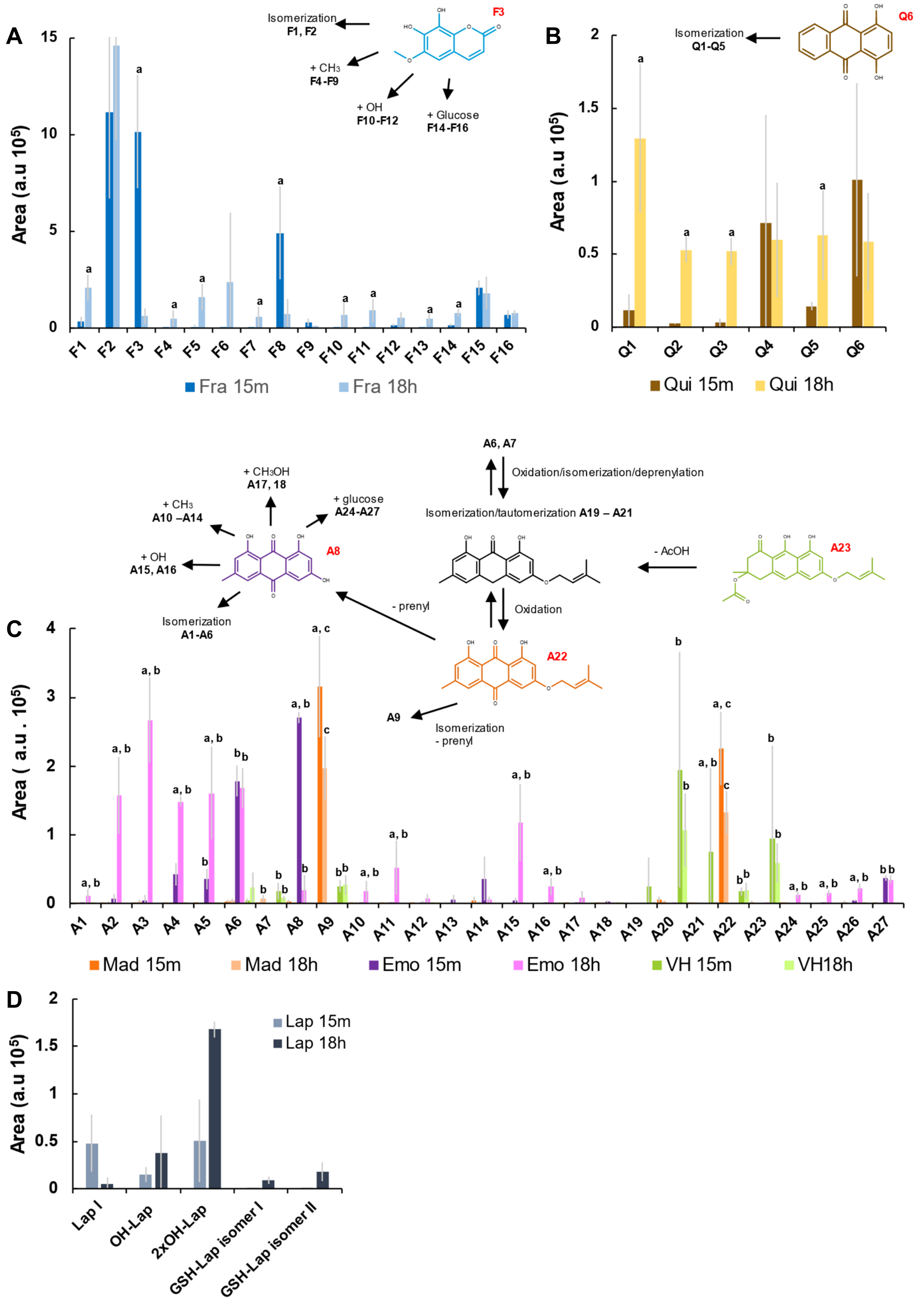

Figure S18. Non-targeted metabolomics of BY-2 cells treated by studied phenolic compounds. Hypothetical metabolization pathways according to reference and related metabolites only detected in ESI/HPLC-QTOF- MS2 analysis of methanolic extracts from BY-2 cells treated 15 min and 18 h with 50 µM of **(A)** fraxetin Fra, **(B)** quinizarin Qui or **(C)** vismione H VH, emodin Emo and madagascine Mad, **(D)** lapachol Lap. Proposed reaction such as hydroxylation (+ OH), methylation (+ CH3), hydroxylation/methylation (+ MeOH), glycosylation (+ Glu) and deprenylation (- prenyl) which may occur in metabolization processes. The thickness of the arrows between each hypothetical reaction relates the proportion of the metabolites found according to the reference (red label) and 54 annotations absent or area under the significant threshold in negative control (< 10 000). Statistical analysis was performed on area from annotated metabolites using Levene with Kruskal-Wallis tests followed by a Dunnett's post-hoc test. Significant differences ($p \leq 0.05$) between area of metabolites from BY-2 cells treated 15 min and 18 h (a) or between treatments with anthranoids (b ≠ c).

2. Supplemental Tables

Table S1. pKa values of pure compounds measured in solution.

	**p*Ka*1**	**p*Ka*2**	**p*Ka*3**
**V**ismione **H** (VH)	7.2 ± 0.2	13.2 ± 0.2	na
**Emo**din (Emo)	7.10 ± 0.06	12.70 ± 0.06	> 13
**Mad**agascine (Mad)	11.1 ± 0.1	> 13	na
**Qui**nizarin (Qui)[b]	8.50 ± 0.03	10.65 ± 0.05	na
**Fra**xetin (Fra)	8.2 ± 0.5	> 9.75	na
**Lap**achol (Lap)	6.7 ± 0.1	na	na

[a] From UV-Vis absorption spectrophotometric titrations *versus* pH in EtOH/water 1:1 *v*/*v* with 0.1 M of NaCl; *T* = 25°C. [b] From reference [3]. na = not applicable. Error given as σ with σ = standard deviation.

To find a compromise between the solubility of anthranoids and a high ionic strength as inside the cells, the pK_a values of the selected compounds were measured in EtOH/water 1:1 supplemented with 0.1 M NaCl. The UV-visible absorption spectrophotometric titrations *versus* pH of Fra, Lap, VH, Mad and Emo were performed using an automatic titrator system 794 Basic Titrino (METROHM) with a combined glass electrode (METROHM 6.0234.500, Long Life) filled with 0.1 M NaCl in water/ethanol 50/50 *v*/*v* and connected to a microcomputer (TIAMO light 1.2 program). The combined glass electrode was first calibrated as a hydrogen concentration probe by titrating known amounts of hydrochloric acid (~1.72 or ~ 4.6 × 10^{-1} M from HCl, SIGMA-ALDRICH, puriss pa, >37 %) with CO_2-free sodium hydroxide solution (~ 0.98 M or ~ 1.23 × 10^{-1} M from NaOH, BDH, AnalaR, 98%). The HCl and NaOH solutions were freshly prepared and titrated with sodium tetraborate decahydrate ($B_4Na_2O_7.10H_2O$, FLUKA, puriss, p.a., > 99.5%) and potassium hydrogen phthalate ($C_8H_5KO_3$, FLUKA, puriss, p.a., > 99.5%), respectively, with methyl orange (RAL) and phenolphthalein (PROLABO, purum) used as colorimetric indicators. The GLEE program [7], was applied for the glass electrode calibration (standard electrode potential E_0/mV and slope of the electrode/mV pH^{-1}) and to check carbonate levels of the NaOH solutions used (< 5 %). The autoprotolysis constant of water in water/ethanol 50/50 *v*/*v* medium was fixed at 15.75 [8]. An aliquot of 40 mL of solutions containing 136 μM Lap, 63.2 μM VH, 43.8 μM Emo, 35 μM Mad or 70.9 μM Fra was introduced in a jacketed cell (METROHM) maintained at 25.0(2) °C (LAUDA E200 thermostat). The initial pH was adjusted to ~ 2.9-3.3 with HCl (SIGMA-ALDRICH, puriss pa, >37%) and the titration was then carried out by automatic addition (automatic titrator system 794 Basic Titrino) of known volumes of NaOH solutions (BDH, AnalaR). After each addition (*i.e.* DET method - Dynamic Potential Titration - with a measuring point density of 3), an absorption spectrum was repeatedly recorded using a Varian CARY 50 spectrophotometer fitted with Hellma optical fibres (HELLMA, 041.002-UV) and an immersion probe made of quartz suprasil (HELLMA, 661.500-QX) of 1 cm optical pathway and interfaced (CETRIB) with the potentiometric unit. The spectrophotometric data were then analysed with SPECFIT program [9], which adjusts the absorptivities, and the stability constants of the species formed at equilibrium. SPECFIT uses factor analysis to reduce the absorbance matrix and to extract the eigenvalues prior to the multiwavelength fit of the reduced data set according to the Marquardt algorithm [10,11].

Table S2. Metabolites identified in non-targeted metabolomic analysis of MeOH extracts from BY-2 cells treated with 50 µM Lap

#	measured *m/z*	Detected Ion	Rt (min)	*Δm/z* [ppm]	mSigma	Molecular Formula	Annotation	fragments *m/z* (Relative intensity %)
L1	243,1017	$[M+H]^+$ [M+Na]+	8.98	0.42	13.7	$C_{15}H_{14}O_3$	Lap I	205.048 (18), 187.038 (100), 159.043 (80), 131.049 (4.7), 105.033 (3.1), 61.039 (1.8), 43.017 (6)
L2	259,0967	$[M+H]^+$ [M-H2O+H]$^+$, $[M+Na]^+$	7.51	0.82	7.6	$C_{15}H_{14}O_4$	OH-Lap	223.076 (4.4), 195.080 (1.6), 159.044 (2.2), 71.049 (2.6), 61.039 (100), 43.053 (12)
L3	277,1073	$[M+H]^+$ $[M+Na]^+$, $[M-H_2O+H]^+$	8.00	0.667	2.6	$C_{15}H_{16}O_5$	2xOH-Lap	241.085 (24), 219.056 (100), 195.080 (25), 175.038 (47), 69.069 (73)
L4	566,1806	$[M+H]^+$	5.58	-0.315	10.2	$C_{25}H_{31}N_3O_{10}S$	GSH-Lap I*	
L5	566,1804	$[M+H]^+$	7.62	0.24	12.0		GSH-Lap II*	

Exact mass-to-charge ratio (m/z), detected ion, retention time (R_t), m/z ratio of parent, Δm/z (ppm), variation of isotopic ratio (mSigma) and fragment ions were obtained from the UPLC-MS/MS HR chromatograms in positive ionization mode. *The absence of MS/MS fragmentation

Table S3. Metabolites identified in non-targeted metabolomic analysis of MeOH extracts from BY-2 cells treated with 50 μM Fra

#	measured *m/z*	Detected Ion	Rt (min)	*Δm/z* [ppm]	mSigma	Molecular Formula	Annotation	fragments *m/z* (Relative intensity %)			
								m/z	***a***	***b***	***c***
F1	209.0445	$[M+H]^+$	4.88	0.184	4.8	$C_{10}H_8O_5$	Fra I [a]	194.021	48	56	51
F2	209.0444	$[M+H]^+$	5.54	-0.085	3.2		Fra II [b]	166.026	18	23	22
F3	209.0444	$[M+H]^+$ $[M+Na]^+$, $[M+NH_4]^+$	6.00	0.19	4.7		Fraxetin [c]	153.054	14	17	17
								149.023	29	35	33
								135.044	8	12	11
F4	223.0603	$[M+H]^+$	5.51	0.99	10.5	$C_{11}H_{10}O_5$	Me-Fra I [a]	208.036	-	51	35
F5	223.06	$[M+H]^+$	5.81	-0.42	2.9		Me-Fra II [b]	190.026	49	68	51
F6	223.0601	$[M+H]^+$	5.86	-0.26	7.7		Me-Fra III*	179.034	13	19	15
F7	223.0603	$[M+H]^+$	6.08	0.835	2.3		Me-Fra IV*	162.031	48	49	47
F8	223.0602	$[M+H]^+$ $[M+Na]^+$	6.47	0.268	5.0		Me- Fra V [c]	149.023	7	3	-
F9	223.06	$[M+H]^+$	6.70	-0.56	16.5		Me- Fra VI*	107.049	10	3.7	11
								78.046	-	3.5	3.1
F10	225.0394	$[M+H]^+$	4.69	0.18	4.2	$C_{10}H_8O_6$	OH- Fra I [a]	210.016	100	100	100
F11	225.0395	$[M+H]^+$	4.95	0.473	1.5		OH- Fra II [b]	182.021	4.7	-	6.1
F12	225.0394	$[M+H]^+$	5.61	0.18	11.8		OH- Fra III [c]	165.018	7.0	7.0	4.7
								154.026	10	10	10
F13	239.0551	$[M+H]^+$	5.54	0.56	3.1	$C_{11}H_{10}O_6$	MeOH- Fra*				
F14	371.0976	$[M+H]^+$	4.88	0.794	6.8	$C_{16}H_{18}O_{10}$	Glu- Fra I*	209.044	100	100	
F15	371.0975	$[M+H]^+$	5.48	0.592	0.2		Glu- Fra II [a]	194.021	4.2	4.4	
F16	371.0977	$[M+NH_4]^+$, $[M+Na]^+$	5.53	0,831	6.1		Glu- Fra III [b]	145.085	-	4	
								71.049	-	3.22	

Exact mass-to-charge ratio (m/z), detected ion, retention time (R_t), m/z ratio of parent, Δm/z (ppm), variation of isotopic ratio (mSigma) and fragment ions were obtained from the UPLC-MS/MS HR chromatograms in positive ionization mode. [a-c] association between metabolites annotated and their parent and their main fragments detected after fragmentation at different collision energy. *The absence of MS/MS fragmentation or fragments (-).

Table S4. Metabolites identified in non-targeted metabolomic analysis of MeOH extracts from BY-2 cells treated with 50 µM Qui.

#	measured *m/z*	Detected Ion	Rt (min)	*Δm/z* [ppm]	mSigma	Molecular Formula	Annotation	fragments *m/z* (Relative intensity %)					
								m/z	*a*	*b*	*c*	*d*	*e*
Q1	241.0495	$[M+H]^+$	5.86	0.158	1.9	$C_{14}H_8O_4$	Qui I [a]	241.049	100	100	100	100	100
Q2	241.0498	$[M+H]^+$	6.36	1.25	5.7		Qui II*	213.054	20	16	20	18	21
Q3	241.0496	$[M+H]^+$	7.74	0.6	8.9		Qui III [b]	185.059	27	26	24	26	28
Q4	241.0492	$[M+H]^+$	8.3	-1.44	2.0		Qui IV [c]	157.064	21	18	18	18	20
Q5	241.0495	$[M+H]^+$	8.59	0.29	3.6		Qui V [d]	129.069	7.7	5.5	6.4	-	6.8
Q6	241.0496	$[M+H]^+$	10.49	0.021	4.3		Quinizarin [e]	59.049	-	-	-	15.33	-

Exact mass-to-charge ratio (m/z), detected ion, retention time (R_t), m/z ratio of parent, Δm/z (ppm), variation of isotopic ratio (mSigma) and fragment ions were obtained from the UPLC-MS/MS HR chromatograms in positive ionization mode. [a-e] association between metabolites annotated and their parent and their main fragments detected after fragmentation at different collision energy. *The absence of MS/MS fragmentation or fragments (-).

Table S5. Metabolites and fragments identified in non-targeted metabolomic analysis of MeOH extracts from BY-2 cells treated with 50 µM Emo, Mad and VH.

#	measured *m/z*	Detected ion	Rt (min)	*Δm/z* [ppm]	m Sigma	Molecular Formula	Annotation	fragments *m/z* (Relative intensity %)							
								m/z	*a*	*b*	*c*	*d*	*e*	*f*	*g*
A1	271.0600	$[M+H]^+$	5.8	0.06	2.7	$C_{15}H_{10}O_5$	Emo I*	253.049	-	-	-	-	1.4	1.5	1.5
A2	271.0602	$[M+H]^+$	6.17	0.387	2.6		Emo II [a]	229.049	42	43	41	40	19	-	-
A3	271.0601	$[M+H]^+$	7.54	0.03	9.0		Emo III [b]	201.054	23	23	24	23	-	-	-
A4	271.0602	$[M+H]^+$	7.71	0.636	4.8		Emo IV [c]	197.059	-	-	-	-	14	17	17
A5	271.0601	$[M+H]^+$	8.49	0.324	1.1		Emo V [d]	173.059	12	12	13	12	4.4	-	-
A6	271.0601	$[M+H]^+$	9.06	0.56	0.8		Emo VI [e]	169.064	-	-	-	-	-	4.8	4.2
A7	271.0603	$[M+H]^+$	9.99	1.265	15.4		Emo VII *	145.064	3.8	3.6	4.3				
A8	271.0601	$[M+H]^+$	10.38	0.396	9.1		Emodin [f]	115.054	-	1.6	1	1.4	1	1.5	-
A9	271.0602	$[M+H]^+$	11.73	0.747	3.0		Emo VIII [g]	95.013	1.7	1.3	1.4	1.3	-	-	-
A10	285.0757	$[M+H]^+$	7.64	-0.02	9.6	$C_{16}H_{12}O_5$	Me-Emo I [a]	252.042	-	2.6	3.4				
A11	285.0758	$[M+H]^+$	8.42	0.21	9.8		Me-Emo II [b]	242.056	6.5	10.4	10				
A12	285.076	$[M+H]^+$	8.94	1.01	8.7		Me-Emo III*	211.075	6.1	9.9	9.7				
A13	285.076	$[M+H]^+$	9.55	1.025	2.0		Me-Emo IV*	196.052	-	1.9	1.9				
A14	285.0758	$[M+H]^+$	11.01	0.02	3.8		Me-Emo V [c]								
A15	287.0552	$[M+H]^+$	7.4	0.413	2.1	$C_{15}H_{10}O_6$	OH-Emo I	269.044 (9.2), 241.049 (27), 213.054 (10), 199.038 (4.9),							
A16	287.0551	$[M+H]^+$	7.88	0.226	3.5		OH-Emo II*	185.059 (3.2), 157.064 (2.8)							
A17	301.0709	$[M+H]^+$	7.17	0.82	12.2	$C_{16}H_{12}O_6$	MeOH-Emo I [a]	255.065	11.5	10.5					
A18	301.0709	$[M+H]^+$	9.63	0.7	3.6		MeOH-Emo II [b]	227.069	5.3	5.4					
A19	325.1439	$[M+H]^+$	8.85	1.32	27.5	$C_{20}H_{20}O_4$	Mad anthrone I*	257.080	100	100					
A20	325.1435	$[M+H]^+$	10.62	0.571	4.8		Mad anthrone II [a]	239.070	19.8	11.9					
A21	325.1435	$[M+H]^+$, $[M+Na]^+$	11.46	0.2275	10.8		Mad anthrone III [b]	211.075	11.2	9.9					
								69.070	3.6	7.4					
A22	339.1226	$[M+H]^+$, $[M+Na]^+$	11.73	0.92	4.3	$C_{20}H_{18}O_5$	Madagascine	283.060 (8.5), 271.060 (100), 197.059 (3.1), 69.069 (9.1)							
A23	385.1645	$[M+H]^+$, $[M+Na]^+$	10.63	0.512	4.9	$C_{22}H_{24}O_6$	Vismione H	Only $[M+Na]^+$ 347.125 (14), 287.091 (100), 269.080 (26), 245.080 (39), 60.081 (6)							
A24	433.1131	$[M+H]^+$	6.17	0.3	22.4	$C_{21}H_{20}O_{10}$	Glu-Emo I [a]	271.060	100	100	100				
A25	433.113	$[M+H]^+$	7.54	-0.15	17.4		Glu-Emo II*	229.049	4.8	4.3	4.5				
A26	433.113	$[M+H]^+$	8.44	0.505	6.6		Glu-Emo III [b]	85.028	15	11	12				
A27	433.1132	$[M+H]^+$, $[M+Na]^+$	9.03	0.888	5.6		Glu-Emo IV [c]	69.033	3.9	2.3	3.4				

Exact mass-to-charge ratio (m/z), detected ion, retention time (R_t), m/z ratio of parent, $\Delta m/z$ (ppm), variation of isotopic ratio (mSigma) and fragment ions were obtained from the UPLC-MS/MS HR chromatograms in positive ionization mode. [a-g] association between metabolites annotated and their parent and their main fragments detected after fragmentation at different collision energy. *The absence of MS/MS fragmentation or fragments (-).

3. Supplemental references

1. Ossowski, T.; Goulart, M.O.F.; Abreu, F.C. de; Sant Ana, A.E.G.; Miranda, P.R.B.; Costa, C. de O.; Liwo, A.; Falkowski, P.; Zarzeczanska, D. Determination of the PKa Values of Some Biologically Active and Inactive Hydroxyquinones. *J. Braz. Chem. Soc.* **2008**, *19*, 175–183, doi:10.1590/S0103-50532008000100025.
2. da Cunha, A.R.; Duarte, E.L.; Lamy, M.T.; Coutinho, K. Protonation/Deprotonation Process of Emodin in Aqueous Solution and PKa Determination: UV/Visible Spectrophotometric Titration and Quantum/Molecular Mechanics Calculations. *Chem. Phys.* **2014**, *440*, 69–79, doi:10.1016/j.chemphys.2014.06.009.
3. Sedaira, H.; Idriss, K.A.; Seleim, M.M.; Abdel-Aziz, M.S. Use of Quinizarin as a Spectrophotometric Reagent for MgO Content Analysis of Portland Cement and Cement Clinker. *Monatshefte Für Chem. Chem. Mon.* **1998**, *129*, 49–58, doi:10.1007/PL00010104.
4. Nebenführ, A.; Gallagher, L.A.; Dunahay, T.G.; Frohlick, J.A.; Mazurkiewicz, A.M.; Meehl, J.B.; Staehelin, L.A. Stop-and-Go Movements of Plant Golgi Stacks Are Mediated by the Acto-Myosin System. *Plant Physiol.* **1999**, *121*, 1127–1141, doi:10.1104/pp.121.4.1127.
5. Greenspan, P.; Mayer, E.P.; Fowler, S.D. Nile Red: A Selective Fluorescent Stain for Intracellular Lipid Droplets. *J. Cell Biol.* **1985**, *100*, 965–973, doi:10.1083/jcb.100.3.965.
6. Gomord, V.; Denmat, L.-A.; Fitchette-Laine, A.-C.; Satiat-Jeunemaitre, B.; Hawes, C.; Faye, L. The C-Terminal HDEL Sequence Is Sufficient for Retention of Secretory Proteins in the Endoplasmic Reticulum (ER) but Promotes Vacuolar Targeting of Proteins That Escape the ER. *Plant J.* **1997**, *11*, 313–325, doi:10.1046/j.1365-313X.1997.11020313.x.
7. Gans, P. GLEE, a New Computer Program for Glass Electrode Calibration. *Talanta* **2000**, *51*, 33–37, doi:10.1016/S0039-9140(99)00245-3.
8. Fonrodona, G.; Ràfols, C.; Bosch, E.; Rosés, M. Autoprotolysis in Aqueous Organic Solvent Mixtures. Water/Alcohol Binary Systems. *Anal. Chim. Acta* **1996**, *335*, 291–302, doi:10.1016/S0003-2670(96)00329-7.
9. Gans, P. Data Fitting in the Chemical Sciences: by the method of least squares. *Angew. Chem.* **1993**, *105*, 654–654, doi:10.1002/ange.19931050453.
10. Marquardt, D.W. An Algorithm for Least-Squares Estimation of Nonlinear Parameters. *J. Soc. Ind. Appl. Math.* **1963**, *11*, 431–441, doi:10.1137/0111030.
11. Maeder, Marcel.; Zuberbuehler, A.D. Nonlinear Least-Squares Fitting of Multivariate Absorption Data. *Anal. Chem.* **1990**, *62*, 2220–2224, doi:10.1021/ac00219a013.
